# Comparing Blood Sampling Techniques in Canines: A Pilot Study Using Oclacitinib

**DOI:** 10.3390/vetsci12060543

**Published:** 2025-06-03

**Authors:** Emily Ryman, Merilyn Dobbs, Leslie Gabor, Abishek Santhakumar, Brian Cassar, Nidhish Francis

**Affiliations:** 1 Elanco Animal Health, 245 Western Road, Kemps Creek, NSW 2718, Australia; emily.ryman@elancoah.com (E.R.); merilyn.dobbs@elancoah.com (M.D.); leslie.gabor@elancoah.com (L.G.); brian.cassar@elancoah.com (B.C.); 2 School of Dentistry and Medical Sciences, Faculty of Science and Health, Charles Sturt University, Wagga Wagga, NSW 2678, Australia; asanthakumar@csu.edu.au

**Keywords:** canine blood collection, venepuncture, canine catheter, cortisol, pharmacokinetics

## Abstract

Pharmacokinetic studies are critical to assess drug absorption, distribution, metabolism, and excretion in companion animals. Blood collection methods such as direct venepuncture or indwelling catheters could influence pharmacokinetic outcomes and animal welfare. A direct comparison of drug concentrations of two blood sampling methods was investigated in this study to identify any potential differences and their impact on animal welfare. Four canines (male = 3, female = 1) were treated with Apoquel^®^ (oclacitinib 0.4–0.6 mg/kg) and blood samples were obtained via direct venepuncture into the jugular and a cephalically placed catheter. The drug distribution and cortisol concentration were examined over several time points (0.25, 0.5, 1, 2, 4, and 6 h post treatment). Statistical analysis revealed no significant differences (*p* > 0.05) in the concentration of the drug between the two collection methods, indicating that both methods are acceptable in generating reliable results for pharmacokinetic data. Nevertheless, cortisol levels indicated a trend suggesting catheter collection may be associated with reduced stress compared to direct venepuncture (Catheter = 201 ± 91; Direct venepuncture = 208 ± 96. This study provides evidence to use a less invasive blood collection such as via a catheter during intensive bleeding schedules that are required in early drug development, thereby improving the overall welfare for the animal.

## Simple Summary

This study aimed to identify whether different blood sampling methods affect the measurements of drug levels and stress in dogs. For this, the dogs were treated with a drug called Apoquel, a drug commonly used for treating allergic skin diseases in dogs. Two techniques, namely, direct needle draws from a major vein in the neck and collections through a preplaced forearm catheter were compared. With the final four dog participants in the study, blood samples were taken at regular intervals for six hours to track the movement of the drug and measure the stress hormone levels, cortisol. Results showed that both the blood collection methods were equally reliable, but the use of the catheter tended to reduce the stress associated with blood collection. The study highlights how small changes in medical procedures can improve animal welfare without compromising data quality, benefiting both pets and veterinary care standards.

## 1. Introduction

The demand for companionship from animals is on the rise and therefore the need to expand and better understand their healthcare management has also increased. As a result, animal research plays a pivotal role in current society, with a key aspect of veterinary healthcare focusing on developing drugs for companion animals. Pharmacokinetics (PK) studies are often used in research and development (R&D) procedures to understand the absorption, distribution, metabolism, and excretion of a drug in the body over a defined time [1]. Pharmacokinetic studies are paramount in early drug development as the pharmacological response, and length of action are often heavily scrutinized [1]. These studies play a vital role in the drug registration and approval process as the analysis of PK concentrations helps assess the safety and effectiveness of the drug.

A disadvantage of pharmacokinetic models is the variation of results depending on the administration route and what molecules are used to deliver the active. In early research, a variety of different systems may be identified for delivery of the drug and therefore data will need to be generated for each of these to determine which is most suitable and most optimal [1]. This can result in a large number of studies being required in early development before investigation of the drug can occur.

An important method of PK is the absorption of a drug which can be examined through the analysis of blood collected throughout the period of interest, usually during the duration of the drug’s action and graphing the concentration in the plasma [1].

While conducting animal research investigations, one should strike a good balance between sourcing reliable data and maintaining the welfare of animals. Thus, the approaches used for blood collection during research need to be examined [2].

Generally, for animals, there are two methods routinely used for blood collection: direct venepuncture or an indwelling catheter [3]. Blood collection from direct venepuncture is typically performed with a needle and syringe, inserted directly into the jugular or cephalic vein (saphenous vein is sometimes used but not as common) and the blood sample is aspirated into the syringe [4]. The blood sample is then transferred into the relevant blood tubes for further analysis. Similarly, blood collection from an indwelling catheter typically involves flushing the catheters with a solution to maintain patency, followed by drawing the blood samples onto the syringe. It should be noted that the standard method for the maintenance of catheter patency is not suitable, particularly for PK studies. The catheter is usually flushed with a heparinised solution, that helps to prevent clotting or fibrin build-up within the catheter lumen and therefore prolong the successful use [5]. However, the use of heparinised solutions is often avoided in the development of novel drugs due to the risk of drug-to-drug interactions which may lead to skewed results or safety problems [6]. Therefore, saline-only flush is predominantly relied upon for maintaining catheter patency for PK studies.

Another significant consideration in animal research is ensuring that animal welfare is sustained. A measure of this is ensuring animals have the least exposure to stressful triggers wherever possible, which can be quantified by evaluating the concentration of an important biomarker of stress, cortisol in the blood [7]. The current literature demonstrates that blood collection via direct venepuncture can cause higher levels of cortisol concentration, likely due to the immediate stress of blood collection [3]. However, additional studies [8] provided evidence that human interaction has a positive impact on reducing the concentration of cortisol in the dog’s plasma; therefore, blood collection procedures and handling have considerable scope to be further refined to help minimise blood collection-associated stress in the animal. Many studies have evaluated cortisol concentrations, specifically in dogs; however, a direct comparison of stress associated with both blood collection methods has not yet been investigated.

For this study, oclacitinib (trade name: Apoquel^®^; Zoetis Inc., Pasippany, NJ, USA) was selected as the marker drug due to its rapid absorption, wide safety of margin, and readily available manufacturing details and qualification methods [9]. Oclacitinib is a selective JAK inhibitor (Janus kinase inhibitor), an immune-modulating medication that can inhibit the activity of enzymes from the Janus kinase family [10]. It is used in the treatment of canine pruritus associated with allergic dermatitis and atopic dermatitis in canines [10].

Whilst both methods of blood collection (direct venepuncture and via catheter) are widely approved and used in several research studies, there is very limited research that has aimed to make a direct comparison of the two methods in the context of animal welfare. This study aimed to directly compare the drug concentrations of both methods and the impact these methods may have on blood cortisol, a known biomarker of stress. Gaining a deeper understanding of the impacts of blood collection methods on animal stress levels will allow researchers to make informed decisions on their approaches to blood collection.

## 2. Materials and Methods

### 2.1. Study Subjects and Inclusion Criteria

This pilot study was conducted under an Animal Research Authority issued by the Yarrandoo Animal Ethics Committee (AEC) on 14 December 2022 (Ethics study number ELA1229). The study was performed at Yarrandoo, the Elanco Animal Health R&D Australia site. The study initially selected 10 dogs (6 males and 4 females) from the colony available on site to be enrolled and acclimatised to a temperature-controlled facility for seven days (day 7 till day 0) before treatment. The criteria for inclusion are as follows: being in good health and physical condition; at least 12 months of age on the day of treatment; and had not received any other investigational products within the last month. Additionally, only purebred beagles were used, with no specific requirements around males or females, desexed or entire status, as long as females were not pregnant or lactating.

Throughout the study, the dogs were examined at least once daily on their general health status and any abnormal findings were documented. Additionally, during the acclimatisation period, all 10 dogs underwent a veterinary examination, initial blood collection via direct venepuncture to establish a baseline, and trialling of having their front limb bandaged. This trialling was performed on three separate occasions to investigate any dogs who may not tolerate having their limbs bandaged which may then cause damage to the catheter and incorrect data. The toleration was measured by any incidence of chewing or scratching at the bandage, as well as being left alone with the bandage and if it was still present after 10 min. The main indicators for suitability were if the dogs were to chew at the bandages, would scratch or remove their bandage or did not tolerate it very well with behaviours such as stiffness in the limb or unwillingness to move/utilise it, elevated anxiety, distress, agitation, and discomfort. The animals who best tolerated this experience during each trialling (*n* = 4) were selected for the study (3 males and 1 female). The remaining (*n* = 6) showed signs of intolerance and were indicated as spares and remained in acclimatisation until completion of the study.

This trialling was performed after the dogs had their afternoon food ration. During this period, they would be individually housed for feeding and would cause minimal disruption to their daily activities. Throughout all other periods (including overnight housing and outside physical activity during the day) besides morning feed rations, the dogs were group housed in their compatible pairs/groupings. All dogs were fed with commercially available dry food twice daily, and food enrichment items were provided throughout the study, with an exclusion period of 12 h before treatment and 4 h post treatment. All dogs were returned to the colony after the completion of study.

### 2.2. Drug Administration

On the first day, all dogs including both the allocated study participants and the reserve animals, were kept without food and maintained in a fasted state. Prior to treatment, the four dogs that met the inclusion criteria were catheterised by appropriately trained personnel. All dogs received the same treatment dosage and underwent identical blood sampling procedures as a within-subjects cross-sectional study.

The procedure included clipping the hair on the limb around the insertion site, cleaning the insertion site with isopropanol, inserting the catheter to prevent blood spillage, and securing the catheter to the limb to prevent any damage to the cephalic vein and minimise the risk of being removed. Once inserted, the catheter was then flushed with 3 mL of sterile 0.9% sodium chloride to confirm the successful insertion of the catheter and adequate flow. Once catheterised, the dogs were given a single tablet via oral treatment of Apoquel^®^ (0.4–0.6 mg/kg—registered dose) as per the registered administration instructions. Oral administration was spaced with 5 min between each animal, to allocate enough time for treatment and blood sampling between each animal. The administration involved placing the tablet at the back of the animal’s mouth and immediately followed by 5 mL of water from a syringe to help promote swallowing. Care was taken to ensure the entire dose was consumed and there was no loss of product; the mouths were also checked following consumption to confirm this. The administration of Apoquel drug was successful for all animals with no loss of product. The timing of oral administration for each dog was documented to help determine the exact timing of the subsequent blood collections and were collected as closely to the indicated time as possible.

Additionally, a post-treatment observation was performed one hour (±5 min) after administration to ensure there were no unexpected reactions or regurgitation that may have occurred. If the dog did regurgitate the drug, then redosing did not take place; however, this or any other observations were documented. No regurgitation of the drug was observed in any of the dogs.

### 2.3. Blood Sampling Methods

The predetermined blood sampling points were 0.25, 0.5,1, 2, 4, 6, and 8 h post treatment. These time points were determined through a review of the literature and industry requirements and were deemed most suitable to provide a sufficient PK curve, capturing all four phases of the drug within the system during research and development phases of novel drugs [9].

At each time point, up to 6 mL of blood was collected across both collection methods (direct venepuncture from the jugular vein, catheter collection from the cephalic vein). The first sample was collected from the catheter via the push–pull method, which involved an initial 1.05 mL removed and reinfused 3 times in a separate syringe. Immediately following this, the collection syringe aspirated the 3 mL sample which was then allocated into separate tubes with approximately 1 mL placed in an EDTA tube for PK analysis and the remaining 2 mL was placed in a serum tube for cortisol analysis. The second sample was then collected as closely as possible with a needle and syringe via direct venepuncture and the sample was divided as per the catheter samples into EDTA and serum tubes. The catheter was then flushed with up to 3 mL as described above. The vein used for direct venepuncture had pressure applied for approximately 30 s to prevent any damage to the vein, and after flushing the catheter, vet wrap was reapplied to the limb to prevent any contamination from the dogs licking the site or any damage to the catheter. The EDTA tubes were placed on ice and the serum samples at room temperature and were transferred to the lab within an hour of collection.

No catheters were removed by the animals and no signs of additional stress or discomfort towards the catheter placement or bandaging were noted and no other adverse events were reported. One catheter (animal 46128) was removed immediately prior to the 6 h time point due to difficulty collecting a sample; however, all other animals had their catheters removed after the 6 h time point due to increased resistance when sampling.

### 2.4. Specimen Preparation and Analysis—Oclacitinib Concentration

The blood samples were collected into EDTA tubes and processed to plasma by being centrifuged at 3000 g for 15 min at 4 °C and then placed into a −20 °C freezer for later analysis using a validated liquid chromatography-tandem mass spectrometry (LC-MS/MS) method [11]. The samples were thawed at room temperature and briefly mixed prior to the extraction process. Separate stock solutions were prepared for the calibration and quality control (QC) solution by dissolving the analyte oclacitinib in Dimethyl sulfoxide (DMSO) to a concentration of 1 mg/mL. Calibration and QC working standards were then prepared in 50% acetonitrile_aq_ at varying concentrations with the lower limit as 0.5 ng/mL and the upper limit as 500 ng/mL [11]. The isotonic working solution was 15 ng/mL baricitinib in 0.1% (*v*/*v*) formic acid in acetonitrile.

Once all samples were prepared, a Phree phospholipid removal plate was placed over a 96-well plate and the blank samples were added to the required wells. For the rest of the wells, baricitinib (15 ng/mL) in 0.1% formic acid in acetonitrile was added and then the relevant calibration, QC, and study samples were added. The plate was vortexed and filtered using the manifold eluting into the 96-well plate and water was added to all samples. The plate was mixed briefly and then sealed prior to injection onto the LC-MS/MS into a Phenomenex Kinetex 2.6 µm BP 50 × 2.1 mm column. The mobile phase was A: 0.1% formic acid in water and B: acetonitrile [11] with a 0.4 mL/min flow rate. The analyte and internal standards were then eluted over a 4 min period from 10% to 100% [11].

The data were then processed, and calibration curves were generated using 1/x^2^ weighted linear regression of the analyte peak area, with a QC acceptance of 100% ± 30% accuracy and ≤30% precision.

### 2.5. Specimen Preparation and Analysis—Cortisol Samples

The blood tubes with cortisol samples were left at room temperature for approximately 2 h to allow enough time for the blood to clot and were then processed in the centrifuge at 2000× *g* for 12 min at room temperature. Once the serum separated, the samples were stored at −80 °C until analysis. Prior to analysis, the samples were removed from the freezer and allowed to thaw completely until they reached room temperature. The cortisol levels were measured using a species-specific canine cortisol ELISA Kit (CUSABIO, Cat. no.CSB-E14303c) according to the manufacturer’s instructions.

### 2.6. Statistical Analysis

The sample size was selected based on the availability of resources and was supported by the literature with similar sample sizes [4,12]. Data were collected and analysed in two programs, Analyst^®^ (Sciex, Mount Waverley, VIC, Australia) 2019, version 1.7.1 for Windows 10, and Thermo Scientific^TM^ (Waltham, MA, USA) Watson LIMS^TM^ version 7.6. A two-way analysis of variance (ANOVA), mixed-effects model with Tukey’s post hoc analysis was performed using GraphPad Prism^®^ 10 2023, version 10.0.3 for Mac. An alpha error of 0.05 was assumed and a statistical significance was established when *p* < 0.05. Statistical interactions that were deemed significant were included in the analysis where possible.

## 3. Results

### 3.1. Blood Drug Concentration

**Figure 1:**
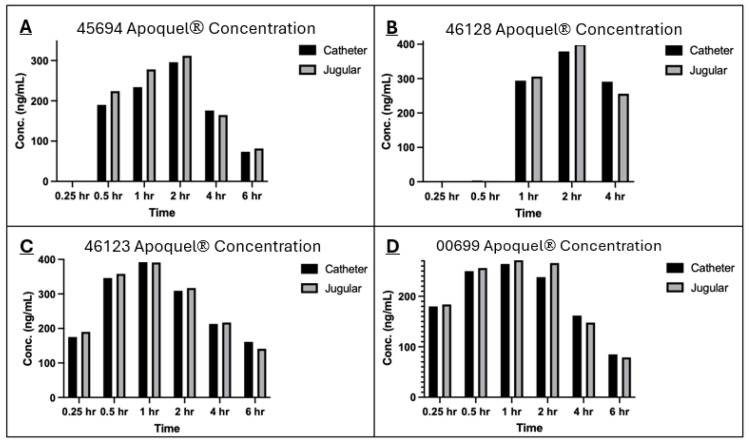
Drug concentrations of Apoquel with both collection methods for each individual animal (**A**–**D**). The drug Apoquel concentration in the blood after oral administration was measured at 0.25, 0.5, 1, 2, 4, and 6 h time points. The blood for analysis was either collected by direct venepuncture or indwelling intravenous catheter.

**Figure 2:**
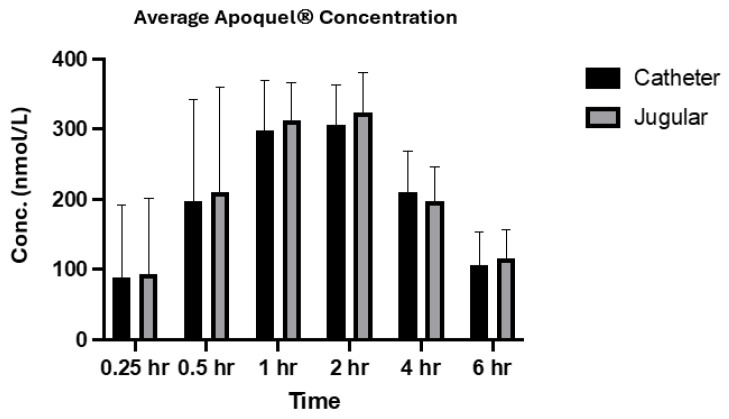
The average drug concentration of Apoquel on all animals (*n* = 4, SD) with both collection methods. Two-factor ANOVA (mixed-effects model) with Tukey’s post hoc analysis was also performed to identify the source of variation between the two collection methods, with a correlation value of 0.99 (*p* = 0.81).

The concentration of Apoquel^®^ was measured using two collection methods (via a catheter in the cephalic vein and direct venepuncture from the jugular vein) at 0.25, 0.5, 1, 2, 4, and 6 h, with the exclusion of one animal only collected (animal # 46128) until the 4 h time point due to the catheter requiring removal (Figure 1). The concentration of Apoquel^®^ began to increase at the 1 h time point and gradually decreased at the 2 h time point. A direct comparison for each animal of the concentration of Apoquel^®^ across all time points, both the jugular venepuncture and cephalic catheter collection methods, show very similar concentrations. No significant difference (*p* > 0.05) in the average drug concentration was noted between the two collection methods (Figure 2).

### 3.2. Cortisol Analysis

**Figure 3:**
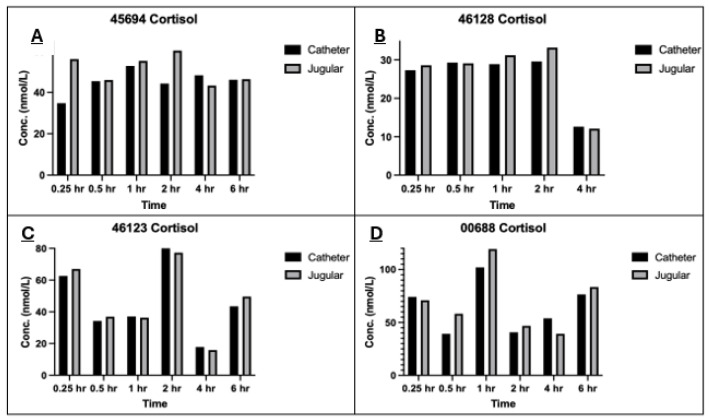
Changes in cortisol concentration over time between collection methods for each animal (**A**–**D**). Cortisol analysis was measured at 0.25, 0.5, 1, 2, 4, and 6 h time points. The blood for analysis was either collected by direct venepuncture or indwelling intravenous catheter.

**Figure 4:**
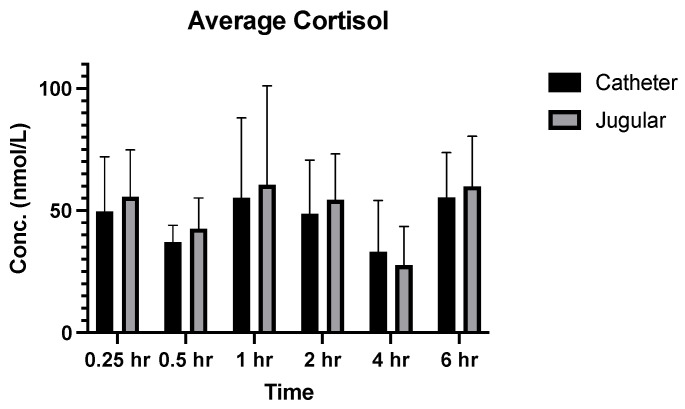
Average cortisol concentration (*n* = 4, SD) over time for both collection methods. Two-factor ANOVA (mixed-effects model) with Tukey’s post hoc analysis was also performed to identify the source of variation for cortisol levels between the two collection methods.

The cortisol concentration was measured at each time point (0.25, 0.5, 1, 2, 4, and 6 h), except for the same animal (animal #46128) as described above, for both jugular venepuncture and catheter collection methods to make direct comparisons (Figure 3). For each animal, there were individual changes in cortisol concentrations. There was no significant difference (*p* > 0.05) in the average cortisol concentration between the two collection methods (Figure 4). Additionally, the data showed a difference in average cortisol concentrations across each period of the study, however, this did not follow a pattern.

## 4. Discussion

The primary aim of this cross-sectional correlation study was to compare the concentration results between two different blood sampling techniques: direct venepuncture and indwelling catheter. Also, the study aimed to evaluate the cortisol concentrations throughout the blood collection time points to understand the stress associated with these procedures. Results showed that there was no significant difference between the two blood collection techniques as expected. This indicates that where applicable, either of the blood collection methods can be used for future pharmacokinetic studies. However, a trend of reduced cortisol levels was observed with the use of indwelling catheter compared to direct venepuncture. The data from this study will be used to guide the methods used in future pharmacokinetic studies in particular research and development (R&D) environments, thereby improving animal welfare.

Both blood sampling methods are well documented in clinical practice; however, the standard use of heparin solutions for catheter maintenance poses risks in the research and development industry due to limited information about the drug-to-drug interaction of novel treatments and heparinised solutions. A saline-only flush is the safest option for pharmacokinetic studies; however, the evaluation of this maintenance procedure on pharmacokinetic results is not well documented [6]. There is currently no appropriate in vitro model to accurately evaluate the analytes in blood levels between the sampling techniques or to capture the changes in cortisol, therefore, data were collected using a live animal study. Both sampling methods were used at each time point to allow direct comparison within each subject, to eliminate variances in individual response to the drug, and repetition between subjects for statistical significance.

The analysis of oclacitinib concentration generated from both sampling methods showed very similar values across both methods, especially for the later time points (Figure 1 and Figure 2). The correlation coefficient between the two methods within individual subjects and across the population at each time point was greater than 0.9 which indicates a strong positive correlation between each collection method. The results from this study are also in alignment with the absorption results generated from Zoetis Inc. (Pasippany, NJ, USA), a study of the PK profile of Apoquel^®^ which used direct venepuncture as the collection method, further confirming and validating the experimental procedures used in this study. Additionally, as described in other literature [11], the oral administration reached maximum plasma concentration at approximately 1 h post treatment and a half-life concentration at approximately 4 h post treatment, and is reflected within the results of this study across both sampling methods (Figure 2).

Another important finding in the study is the differences in cortisol concentrations observed between sampling methods and over the investigation period. On average, when directly comparing both sampling methods, especially when considering the earlier time points, the concentration of cortisol in catheter-collected samples was lower (Figure 4). These data suggest that there is a decrease in stress response to the catheter-collection method during the initial time periods, which means using this method where applicable will have an advantageous effect on the welfare of the animals when several blood collections are required within a short time frame. However, this trend is not followed in the later time points for all subjects. For instance, at the 4 and 6 h time point, while two of the animals (46123 and 46128) had very similar lower cortisol concentrations, and two other animals (00688 and 45694) had higher cortisol concentrations. The literature shows there may be a few reasons to explain the result. A parallelism study of the circadian rhythm and cortisol concentration in dogs [12] showed that when analysing cortisol concentrations over 48 h, the levels increased through the early afternoon with a peak at approximately 14:00 h and decreased soon after [12]. Other studies have also shown that the rhythmic cycle of cortisol follows a similar dawn-to-dusk pattern with the lowest concentrations occurring at 08:00 h and 20:00 h, reaching a peak in the middle of this cycle at approximately 14:00 h [13]. This would also align with the sampling times of this study, with the 4 h collection being in the early afternoon and the 6 h collection falling after 14:00 h, which may explain the differences in the cortisol data observed in this study. Additional studies that investigated the peak of cortisol after experiencing a stressful situation identified the concentration of cortisol in response to stimulation occurs between 2 and 4 h [14]. The treatment and early sampling points required a lot of handling and movement of the animals which would be a stress-stimulating experience; therefore in response to this period and as expected, a peak in cortisol concentration occurred at the 2 h time point in this study. In future studies, the cortisol concentrations may be more accurately examined if the blood collection methods were completed in separate phases at different times as opposed to both methods at the same time, for example, examining the methods on different days. Additionally, the use of equal numbers of both males and females may provide a better insight into the changing cortisol concentrations as there is a difference between basal cortisol levels between sexes [15]. This could also be further refined by using only castrated/desexed or only entire males as these two different states also have influence over basal cortisol levels [15].

The cross-sectional study technique was used specifically to eliminate the risk of results being skewed by variation and compounding variables may be balanced as each animal will undergo both methods. Additionally, due to the small sample size available of dogs which fit the inclusion criteria, there was no distinct “true control” group and instead, each subject acted as a control for its individual results and these data were further strengthened against data from previous Apoquel^®^ studies. Due to limitations of colony availability and time constraints, an appropriate washout period was not feasible in this study design. In future, a longer quasi-experiment would be advantageous, completed in two phases with all animals receiving the same treatment in a single phase, then a short washout period for the drug, followed by the second phase. This will allow both methods to be evaluated and could potentially remove additional variables that could impact the cortisol such as the stress of animals simultaneously experiencing both collection methods. Additionally, increasing the number of animals enrolled initially within the study would help prevent the bias that may be caused by a small sample size due to the limitation of the inclusion criteria. Furthermore, the positive results from this study can be used as evidence to create a more robust training program which introduces dogs to bandages around their limbs from a young age and therefore increasing the amount of animals in the colony who can tolerate this procedure.

Another limitation of the study was maintaining the patency of the catheter for the required investigational period. In clinics routinely, catheters used for blood collection are predominantly connected to IV drips and other infusion methods over longer time periods [4]. This is notable as the constant positive pressure from infusion therapy protects the lumen from blood reflux which, as a result, will prevent any build-ups, clotting, or occlusions [16]. The lack of positive pressure in this study was evident at the later time points (6 and 8 h), where samples were unable to be aspirated due to blockages. During these time points, if samples were difficult to collect, then the catheter was removed for welfare and safety measures, and upon removal, build-up of fibrin and small blots were visible. For future studies, positive pressure-locking techniques could be implemented to help maintain the patency of catheters over time by maintaining continuous pressure within the catheter line to prevent the backflow of blood and any potential buildup of blood clots or occlusions. This is achieved by ensuring the pressure within the catheter line is higher than the venous pressure, which will therefore prevent any occlusion from entering the catheter. This does, however, have additional risks of pushing potential blood clots back into the vein and therefore into circulating blood. Therefore, this procedure may not commonly be used or require additional care and observation of the animal. Furthermore, the catheter size may also be varied based on the size of the dogs selected for the study and may potentially help prolong the patency of the catheter.

This study has demonstrated that there are advantages and disadvantages to collecting blood via an indwelling catheter. This can be advantageous in research with early intensive bleeding periods (e.g., multiple points in one hour) as it requires only one puncture to the cephalic vein and can be used to generate multiple data points. The cortisol results provide evidence that this may have a beneficial impact on the welfare of the animal.

However, there are some disadvantages to the aforementioned particular method, including the difficulty in catheter patency management and acclimatising animals to the procedure. Early training of animals may provide a better tolerance to the procedure and a variety of different techniques may be used to improve collection via the catheter such as sizing and positive-pressure locking. However, for bleed schedules that are less intensive (e.g., 1 h, 2 h, 4 h, etc.) then direct venepuncture may be more suitable as the patency of the catheter deteriorates as the time between bleeds increases and can therefore result in difficult blood collection.

As both methods provide similar concentrations of a drug, either method can be utilised depending on other research factors such as suitability of animals and intensity of the bleed schedules.

## 5. Conclusions

When directly compared, the concentration of the drug from the catheter collection and the direct venepuncture method yielded similar results within an acceptable range. Therefore, the results from this pilot study suggest that either of the blood collection methods (direct venepuncture and indwelling catheter) would be justifiable to generate accurate pharmacokinetic results. Additionally, the use of a catheter for sample collection may have a beneficial impact on animal welfare, generating lower concentrations of cortisol in comparison to alternative methods during the early investigational periods.

## Data Availability

The data that support the findings of this study are available from Elanco Animal Health upon reasonable request and with their permission.

## Abbreviations

The following abbreviations are used in this manuscript:

PK	Pharmacokinetics
R&D	Research and Development
LC-MS/MS	Liquid|Chromatography tandem Mass Spectrometry
QC	Quality control
DMSO	Dimethyl sulfoxide
ANOVA	Analysis of Variance
JAK	Janus kinase
